# Consequences of access to water from managed aquifer recharge systems for blood pressure and proteinuria in south-west coastal Bangladesh: a stepped-wedge cluster-randomized trial

**DOI:** 10.1093/ije/dyaa098

**Published:** 2020-07-12

**Authors:** Abu Mohd Naser, Solaiman Doza, Mahbubur Rahman, Leanne Unicomb, Kazi M Ahmed, Shuchi Anand, Shahjada Selim, Mohammad Shamsudduha, KM Venkat Narayan, Howard Chang, Thomas F Clasen, Matthew O Gribble, Stephen P Luby

**Affiliations:** 1 Gangarosa Department of Environmental Health, Rollins School of Public Health, Emory University, Atlanta, GA, USA; 2 Hubert Department of Global Health, Emory Global Diabetes Research Center, Rollins School of Public Health, Emory University, Atlanta, GA, USA; 3 International Centre for Diarrhoeal Disease Research, Bangladesh (icddr, b), Dhaka, Bangladesh; 4 Department of Geology, University of Dhaka, Dhaka, Bangladesh; 5 Division of Nephrology, School of Medicine, Stanford University, Stanford, CA, USA; 6 Department of Endocrinology, Bangabandhu Sheikh Mujib Medical University, Bangladesh; 7 Institute for Risk and Disaster Reduction, University College London, London, UK; 8 Department of Geography, University of Sussex, Brighton, UK; 9 Department of Biostatistics and Bioinformatics, Rollins School of Public Health, Emory University, Atlanta, USA; 10 Department of Epidemiology, Rollins School of Public Health, Emory University, Atlanta, GA, USA; 11 Division of Infectious Diseases and Geographic Medicine, Stanford University, Stanford, CA, USA

**Keywords:** Managed aquifer recharge, drinking-water salinity, blood pressure, stepped-wedge cluster-randomized trial

## Abstract

**Background:**

Drinking-water salinity has been associated with high blood pressure (BP) among communities in south-west coastal Bangladesh. We evaluated whether access to water from managed aquifer recharge (MAR)—a hydrogeological intervention to lower groundwater salinity by infiltrating rainwater into the aquifers—can reduce community BP.

**Methods:**

We conducted a stepped-wedge cluster-randomized trial with five monthly visits between December 2016 and April 2017 in 16 communities. At each visit following baseline, four communities were randomized to access MAR water. Systolic BP was the primary outcome, measured during each visit using Omron^®^ HEM–907 devices. We also measured participants’ 24-hour urinary sodium and households’ drinking- and cooking-water salinity each visit. We used multilevel regression models to estimate the effects of MAR-water access on participants’ BP. The primary analysis was intention-to-treat.

**Results:**

In total, 2911 person-visits were conducted in communities randomized to have MAR-water access and 2834 in communities without MAR-water access. Households without MAR-water access predominantly used low-salinity pond water and 42% (range: 26–50% across visits) of households exclusively consumed MAR water when access was provided. Communities randomized to MAR-water access had 10.34 [95% confidence interval (CI): 1.11, 19.58] mmol/day higher mean urinary sodium, 1.96 (95% CI: 0.66, 3.26; *p* = 0.004) mmHg higher mean systolic BP and 1.44 (95% CI: 0.40, 2.48; *p* = 0.007) mmHg higher mean diastolic BP than communities without MAR-water access.

**Conclusions:**

Our findings do not support the scale-up of MAR systems as a routine drinking-water source, since communities that shifted to MAR water from the lower-salinity pond-water source had higher urinary sodium and BP.

**Key Messages**

- Saltwater intrusion has contributed to groundwater salinity in many coastal areas of the world.
- The managed aquifer recharge (MAR) system—a hydrogeological intervention to lower groundwater salinity by infiltrating rainwater and pond water into the aquifers—can provide access to low-salinity drinking water.
- We evaluated the health impacts of the routine reliance on MAR systems for drinking water in saltwater intrusion affecting south-west coastal Bangladesh by implementing a stepped-wedge cluster-randomized trial.
- We found that access to MAR water increased population sodium intake and blood pressure.
- Our findings do not support the scale-up of MAR systems as a routine drinking-water source.

## Introduction

Increased groundwater salinity has affected fresh drinking water availability in many coastal communities across South and Southeast Asia.^1^^,^^2^ Coastal aquifer salinity will increase due to increased groundwater extraction to meet agricultural and municipal demand and global climate change, such as sea-level rise.^3^ The south-west coastal region in Bangladesh experiences saltwater intrusion from anthropogenic activities and global climate change, including unplanned saltwater-shrimp cultivation,^4^ decreased upstream river flow,^5^ frequent cyclones^3^ and sea-level rise.^6^ Coastal aquifer salinity has contributed to drinking-water scarcity in south-west coastal Bangladesh.^7^ People in Bangladesh mainly drink groundwater—but many aquifers in south-west coastal Bangladesh are highly saline due to saltwater intrusion. The salinity-induced drinking-water scarcity in south-west coastal Bangladesh occurs during the dry season when there is little rainfall.^8^^,^^9^

**Figure 1:**
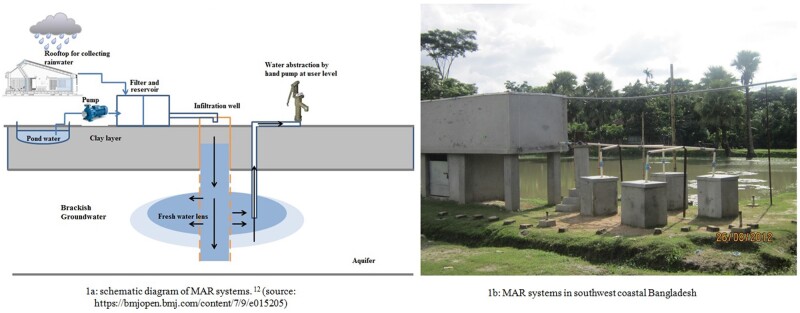
Managed aquifer recharge (MAR) systems in south-west coastal Bangladesh.

During the wet season, communities in south-west coastal Bangladesh drink rainwater.^10^ Coastal communities in Bangladesh have adopted several approaches to secure low-salinity water during the dry season, including rainwater harvesting and pond sand filtration systems.^11^ Although pond water is not commonly used as a water source in other parts of Bangladesh, communities in south-west coastal Bangladesh also drink pond water during the dry season.^12^ Nevertheless, neither harvested rainwater nor pond water is a climate-resilient water source, since their year-round availability is not guaranteed. Ponds in south-west coastal Bangladesh are often inundated by tidal surges and cyclones, which makes pond water unsuitable for drinking. The University of Dhaka Department of Geology, the Government of Bangladesh Department of Public Health Engineering and UNICEF Bangladesh have installed managed aquifer recharge (MAR) systems to provide access to low-salinity drinking water during the dry season in three south-west coastal districts. MAR systems increase the quantity of groundwater by artificially enhancing aquifer recharge and are used in many settings globally to address concerns about water quality and scarcity.^13^ In south-west coastal Bangladesh, MAR systems were initially piloted as a climate-resilient water supply option by infiltrating the rainwater and pond water through sand filtration into the brackish aquifers to create a lens of low-salinity water on top of the brackish aquifers (Figure 1).^14^ MAR systems promise a year-round drinking water supply that is both protected from evaporation and resilient to tidal storms and cyclones, since freshwater infiltration and storage occur at the groundwater level.^15^^,^^16^ A pilot study in coastal Bangladesh evaluated MAR systems installed in 2009 for the feasibility of aquifer recharge, the water-recovery rates and the quality of the recovered water.^14^^,^^15^ This pilot study found MAR water had lower salinity than the pre-treatment brackish groundwater and the water-recovery rate was satisfactory. Based on these findings, the MAR-implementing partners decided to scale up MAR systems in south-west coastal Bangladesh as routine drinking-water sources by constructing 80 new MAR systems in the region by 2014.^15^

Observational studies conducted between 2009 and 2011 in south-west coastal Bangladesh reported that study participants predominantly consumed brackish groundwater and drinking brackish groundwater was associated with high sodium (Na^+^) intake and high blood pressure (BP) among the adults and gestational hypertension among pregnant women.^17^^,^^18^ MAR systems are designed to reduce the intake of ions through drinking water compared with brackish groundwater, but it remains unclear whether drinking water from MAR systems can lower BP or protect against kidney dysfunction.^19^^,^^20^ MAR systems are community-level interventions. Once these systems demonstrated successful salinity reduction below 2 mS/cm, the implementers planned to provide continued MAR-water access to the communities. We took advantage of this roll-out plan for the 80 MAR systems and implemented a stepped-wedge cluster-randomized controlled trial to assess whether MAR systems reduce BP and proteinuria in the population, thereby justifying further scale-up in south-west coastal Bangladesh. We hypothesized that participants switching from consuming brackish groundwater water to lower-salinity MAR water would have lower Na^+^ intake and reduced BP and proteinuria.

## Methods

### Study design, sample size, enrolment and baseline

MAR systems require the infiltration of rainwater and pond water for 2–3 years prior to becoming operational.^15^ Communities were eligible if MAR-system implementers decided that a MAR system was ready for roll-out by November 2016 after water infiltration and MAR-water access was yet to be provided to communities. We calculated a sample size of 16 communities in three districts (Khulna, Satkhira and Bagerhat) of south-west coastal Bangladesh (Supplementar*y* Figure 1, available as Supplementary data at *IJE* online). We initially calculated 1396 total participants needed for a simple unadjusted trial to demonstrated 3-mmHg mean systolic BP difference among the exposed and unexposed groups (effect size = 3, SD = 20 for both groups, 80% power and 5% type I error). We considered 28 households per community or cluster and an average of 2.2 participants (≥20 years old) per household to calculate 3.04 as the design effect for clustering using the formula by Hussey and Hughes.^21^ Therefore, the total sample size was 1396 × 3.04. We further inflated the sample size by 10% considering loss to follow-up. We then calculated that a total of 16 clusters was required considering a fixed cluster size of 60 (28 × 2.2).^22^ We finally calculated that five steps or visits were required by dividing the total sample size by the cluster size and cluster number.

Prior to December 2016, none of the 16 communities had access to MAR water. Before the trial commenced, research assistants visited these 16 communities to list households and household members at least 20 years of age. Some household members (*N* = 45) reported that they occasionally consumed MAR water during the infiltration phase prior to official access when their households faced drinking-water scarcity. Households were eligible for enrolment if they reported that they had never consumed MAR water and expressed willingness to consume MAR water during the study timeline. Research assistants returned to the eligible households to seek informed written consent from the household head and all household members over 20 years old. The study design, rationale, sample-size calculation and site selection procedure have been reported in detail elsewhere.^23^ The evaluation was led by the International Centre for Diarrhoeal Disease Research, Bangladesh (icddr, b) and the study was approved by the human subject committees of icddr, b (PR-15096). The trial was registered at ClinicalTrials.gov (NCT02746003).

### Randomization and masking

Communities gained access to MAR water, in a randomized order, at some time during the second to fifth visits or steps. Using computer-generated random numbers, we randomly selected the four communities that would be provided with access to MAR water in each step following the first (baseline) step. The randomization schedule was assigned by a co-investigator at Emory University who was not involved in the fieldwork. The study could not be blinded so there was no concealment from the participants. In the first (baseline) step, none of the communities had access to MAR water. Once access to MAR water was allowed, communities had continued access. Deviating from the protocol, three communities (one each in steps 3, 4 and 5) did not gain access to MAR water at the randomly assigned times because of ongoing groundwater-quality problems (such as the presence of sand in the MAR water), which would have made it impractical to provide the water at that time. These three communities were not dropped from the study and consumed water from their usual water sources during the entire trial.

### Intervention and promotion

The intervention consisted of providing access to MAR water at the community level and promoting its use as an alternative to brackish water.^14^ We employed two separate groups of research assistants: one for intervention promotion, and one for data collection. The intervention was promoted by locally-employed community health promoters (CHPs) trained by behavioral change communication experts at icddr, b. CHPs met with household members to encourage them to consume MAR water for drinking and cooking when access was provided. CHPs recommended that household members carry a bottle of MAR water when they were away from the household premises for several hours. CHPs visited households at least once per week to encourage MAR-water use. CHPs were expected to work for an average of 60 hours per month to cover 25–30 households and received a remuneration equivalent to US$30 per month, consistently with the then-prevailing wages.

### Exposure and confounder measurement

The data-collection research assistants used a pre-tested structured questionnaire programmed in the open-source Open Data Kit software (University of Washington, Department of Computer Science and Engineering), installed on Android smart phones to collect data during all visits irrespective of intervention status. Research assistants requested information about the drinking- and cooking-water sources for the visit day and asked whether households had stored drinking or cooking water. If stored water was available, researchers collected the water in a pre-sterile 50-mL conical Falcon™ tube and measured the electrical conductivity using a Hanna Salinity™ meter (Romania, model: H198192, range: 0.0–400 mS/cm, accuracy: ±1%). Electrical conductivity—a commonly used indicator of water salinity—measures how easily electrons pass a certain distance in water and is correlated with all dissolved ions in water.^24^

During the first step, research assistants collected information on demographics and household assets, smoking and physical activity defined by the World Health Organization’s Global Physical Activity Questionnaire.^25^ Participant weight was measured in all visits, but height was measured once. Research assistants also collected information about the addition of table salt during cooking (yes or no), consumption of additional table salt with food (yes or no), alcohol consumption (yes or no), hours of sleep, self-reported disease status (hypertension, diabetes, chronic kidney disease) and pregnancy status of the women.

### Outcome measurement

Systolic BP was the primary outcome. Diastolic BP, mean arterial pressure, pulse pressure and proteinuria were secondary outcomes. Trained research assistants measured participants’ BP between 7.30 am and 2.00 pm at each visit day using an Omron^®^ HEM–907 (accuracy: within ±4 mmHg, Kyoto, Japan) device. Participants were asked to avoid eating, smoking and performing physical exercise within 30 minutes of BP measurement. Participants rested for 5 minutes with arms and back supported in a chair prior to BP measurement.^26^ Appropriately sized cuffs based on upper mid-arm circumference (MUAC) were used (small-sized cuff if MUAC <22 cm; medium-sized cuff if MUAC ≥22 to <32 cm; and large-sized if cuff ≥32 cm). BP was measured three times during each visit and the arithmetic mean was used for analysis.

Research assistants collected 24-hour urine samples from available participants at all visits. Each participant received a 4-L plastic container for 24-hour urine collection and a small plastic container to collect the voided urine. Research staff instructed the participants to discard their first morning urine and start collecting from the second morning void. Participants were instructed to transfer all other voids of the day and night into the 4-L plastic container, including the first void of the next day. Urine samples were transported at 2–8°C to a local field laboratory for chemical analysis of total protein, creatinine and minerals. Urine total protein was measured to detect early kidney dysfunction using a colorimetric method by a semi-auto biochemistry analyser (Evolution 3000, BSI, Italy, coefficient of variation: <1%). Urine creatinine was measured by a colorimetric method (Jaffe reaction).

Urinary Na^+^, K^+^, Ca^2+^ and Mg^2+^ were measured as intermediate outcomes to assess the MAR water–BP relationship. The Direct Ion Selective Electrode method, commonly used in clinical biochemistry laboratories with high agreement to the conventional flame photometer,^27^ was used to measure the urinary Na^+^ and K^+^ with a semi-auto electrolyte analyser (Biolyte2000, Bio-care Corporation, Taiwan, coefficient of variation: ±5%). Urinary Ca^2+^ and Mg^2+^ were measured using the photometric titration method using Evolution3000.

We considered 24-hour urine samples to be complete if the participants’ measured daily urinary creatinine concentrations were within 20% of their predicted daily urinary creatinine concentrations.^28^ Participants’ predicted daily urinary creatinine concentrations were calculated using the Kawasaki formula.^29^

### Statistical analyses

The analyses were intention-to-treat. Data were analysed based on the randomization schedule. Participants’ systolic and diastolic BPs were assumed to follow normal distributions and skewed urine protein was assumed to follow a gamma distribution. We used multilevel linear models to analyse the effect of access to MAR water on the difference in mean BP and multilevel parametric quantile regression models to model the median urinary protein in the group with MAR access relative to the no-access group. All models had three-level random intercepts to account for the multilevel clustering of longitudinal visits of the same person, persons within households and households within communities. Models were estimated by maximum likelihood.^30^ Adaptive quadrature with seven points was used to approximate the maximum likelihood for the urine protein model.^31^ We reported cluster-robust standard errors in all models.^32^ The effect of MAR on community BP could be influenced by the time of MAR-water access, so the pre-specified analysis plan was to adjust for visits or steps as a fixed effect in all models. We sequentially fitted models for each outcome considering combinations of confounders. Model 1 adjusted only for the MAR-water-access step or timing and Model 2 further adjusted for age, sex and body mass index (BMI). Model 3 additionally adjusted for smoking status (never-smoker, current smoker and former smoker), work-related physical activity (vigorous physical activities, moderate physical activity and sedentary activity), marital status (married or unmarried), consumption of additional table salt with food (yes or no), alcohol consumption (yes or no), hours of sleep (<6 hours, ≥6 to <9 hours and ≥9 hours) and household-wealth score. Age and BMI were used as linear continuous variables, whereas other variables were categorical. The household wealth score was derived by principal-component analysis using household asset data for ownership of a refrigerator, television, mobile phones, motorcycle, bicycle, sewing machine, chair, table, wristwatch, wardrobe, wooden cot, motor pump, rice-husking machine, motorized rickshaw and car, and access to electricity.

We also assessed whether participants’ 24-hour urinary Na^+^, K^+^, Ca^2+^ and Mg^2+^ concentrations were affected by MAR-water access. Urinary Na^+^ and K^+^ were modelled using multilevel linear regression, whereas skewed Ca^2+^ and Mg^2+^ were modelled by multilevel parametric quantile regression assuming gamma distributions. All statistical models with 24-hour urine biomarkers as outcomes (urine protein, Na^+^, K^+^, Ca^2+^ and Mg^2+^) were restricted to complete 24-hour urine samples. Analyses were conducted using STATA (version 15) and R (version 3.3.1) statistical software.

The counterfactual water sources included all water sources (pond water, rainwater and groundwater) other than MAR. However, prior to the trial, we presumed households would shift from brackish groundwater to MAR water after MAR-water access. In *post hoc* secondary analyses, we assessed the BP and proteinuria among the MAR-water-drinkers compared with the groundwater-drinkers, consistently with our presumption. We conducted two sensitivity analyses. First, we considered the three communities that did not receive MAR-water access as per randomization as non-MAR sites and evaluated the effect of MAR-water access using the multilevel linear or parametric quantile regression models as described before. In the second sensitivity analysis, we used the restricted cubic splines of age and BMI in the models to evaluate whether such forms influence our estimates. The use of antihypertensive drugs could affect the BP of the hypertensive participants. Therefore, in the second sensitivity analysis, we additionally adjusted all BP models for the use of antihypertensive medications.

### Role of funding sources

The study funder (Wellcome Trust, UK) reviewed and approved the study design but was not involved in enrolment, data collection, data analysis, interpretation, manuscript writing and decision to publish.

## Results

**Figure 2:**
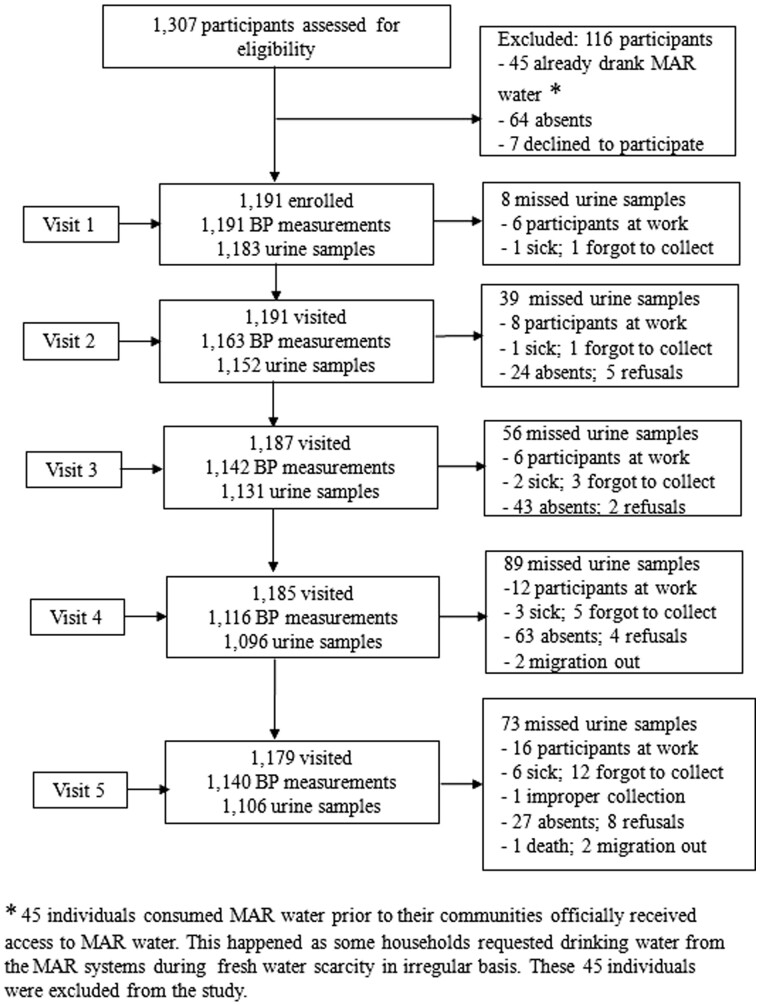
Profile of the managed aquifer recharge (MAR) stepped-wedge cluster-randomized trial.

Research assistants approached 1307 participants during the initial eligibility screening; 1191 were enrolled from 542 households during the baseline visit after obtaining written informed consent (Figure 2). During the five visits, research assistants completed 5752 person-visits for BP measurements and collected 24-hour urine samples from 5668 person-visits. The proportion of participants who were unavailable during the second and subsequent visits were 2.0%, 3.6%, 5.3% and 2.2%, respectively. A few participants refused (*n* = 19, 1.6%) or moved out (*n* = 4) during the study period (Figure 2).

The median age of the participants was 41 (IQR: 31–54) years and the median BMI was 21.8 (IQR: 19.4–24.3) kg/m^2^ (Table 1). Most participants were women (59%), never-smokers (51%), did not report alcohol consumption (97%), were married (96%), of Hindu religion (59%) and consumed additional table salt with food (65%). Among the total participants, 15% reported being hypertensive, 4% being diabetic, 2% reported chronic kidney disease and 1.5% were pregnant (Table 1). All households reported using table salt for cooking.

**Table 1.** Baseline characteristics of the trial participants (*N* = 1191)

Variables	Median (IQR) or % (*n*)
**Age (years)**	41 (31–54)
20 to <30 years	22% (259)
30 to <40 years	26% (309)
40 to <50 years	21% (254)
50 to <60 years	16% (193)
60 to <70 years	10% (120)
≥70 years	5% (56)
**Men**	41% (485)
**Body mass index**	22 (19.38—24.28)
Underweight (<18.5)	16% (186)
Normal weight (18.5 to <25)	64% (765)
Overweight (≥25 to <30)	17% (201)
Obese (≥30)	3% (39)
**Smoker**	
Never	51% (602)
Former	9% (109)
Current	40% (480)
**Participants’ consumption of table salt with food** ^b^	65% (770)
**Reported as hypertensive**	15% (117)
**Reported as diabetic**	4% (49)
**Reported history of chronic kidney diseases**	2% (21)
**Reported as pregnant**	1.5% (18)
**Reported alcohol consumption**	3% (35)
**Sleep hours**	
<6 hours (21%)	21% (251)
6 to >9 hours (66%)	66% (790)
≥9 hours (13%)	13% (150)
**Work-related physical activity** ^a^	
Sedentary	40% (479)
Moderate	31% (370)
Vigorous	29% (342)
**Married**	96% (1143)
**Religion**	
Muslim	41% (494)
Hindu	59% (697)
**Urinary creatinine (mg/day)**	
Male (*N* = 478)	1489 (1161–1883)
Female (*N* = 703)	1161 (933–1432)
**Household-wealth index**	
1st quintile	14% (128)
2nd quintile	22% (116)
3rd quintile	21% (115)
4th quintile	19% (100)
5th quintile	15% (79)

a Measured by the World Health Organization’s Global Physical Activity Questionnaire.

b All households reported to use table salt for cooking. However, 65% participants additionally used table salt with food.

**Figure 3:**
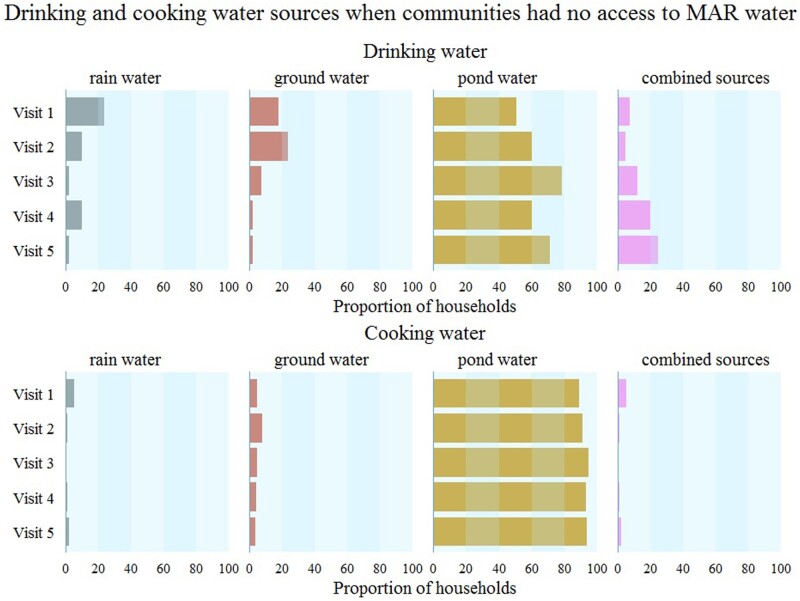
Drinking- and cooking-water sources for communities without access to managed aquifer recharge (MAR) water. Vertical stripes under each source denote 20% of households consuming that water source. Pond water was the predominant source for drinking and cooking when communities had no MAR-water access (counterfactual water sources).

**Figure 4:**
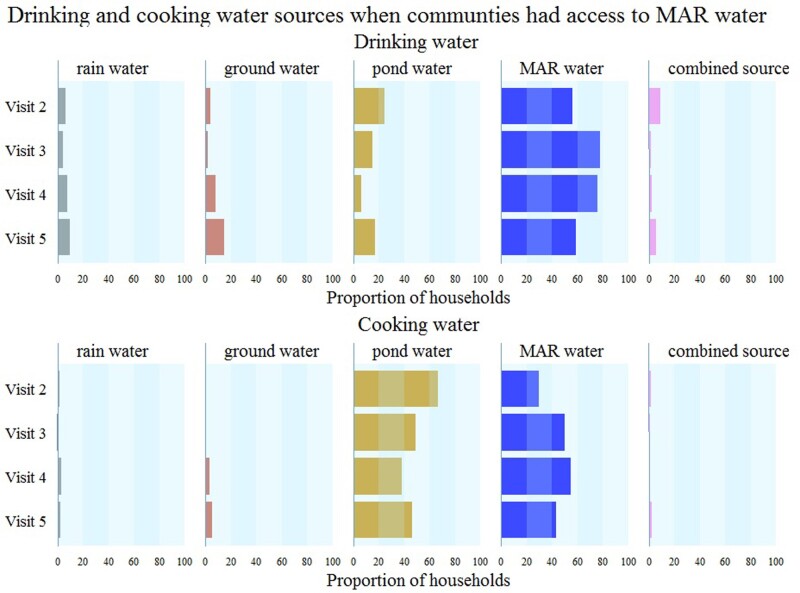
Drinking- and cooking-water sources for communities with access to managed aquifer recharge (MAR) water. Vertical stripes under each source denote 20% of households consuming that water source.

In contrast to our pre-trial assumptions, most households without access to MAR water used pond water rather than brackish groundwater; 60% (range: 51–79%) of households per visit reported exclusive use of pond water for drinking and 90% (range: 89–95%) for cooking (Figure 3). After communities had MAR-water access, households reported that pond-water consumption was 15% (7–25% across visits) for drinking and 47% (range: 38–67% across visits) for cooking (Figure 4), suggesting poor adherence to the MAR intervention. During the baseline visit (Visit 1), 24% of households reported exclusive use of rainwater for drinking (Figure 3), which decreased to ≤10% in subsequent visits (Figures 3 and 4). Rainwater and groundwater were seldom used for cooking.

Combining data from all visits, the median electrical conductivity of rainwater was 71 (IQR: 28–144) µS/cm, pond water was 974 (IQR: 666–1190) µS/cm, MAR water was 1624 (IQR: 1245–2013) µS/cm and brackish groundwater was 3225 (IQR: 2311–4220) µS/cm (Supplementar*y* Figure 2, available as Supplementary data at *IJE* online).

During the study, 2911 person-visits were conducted in communities randomized to have MAR-water access and 2834 person-visits in communities without access. Participants among communities randomized to access MAR water had 1.96 [95% confidence interval (CI): 0.66, 3.26; *p*-value: 0.003] mmHg higher mean systolic BP and 1.44 (95% CI: 0.40, 2.48; *p*-value: 0.007) mmHg higher mean diastolic BP compared with participants from communities randomized to have no access in the full multivariable-adjusted model (Model 3) (Table 2). Participants among communities randomized to access MAR water had 1.62 (95% CI: 0.52, 2.71; *p*-value: 0.004) mmHg higher mean arterial pressure and 0.53 (95% CI: –0.02, 1.07; *p*-value: 0.057) mmHg change in mean pulse pressure compared with participants from communities randomized to have no access in the full multivariable-adjusted model. The ratio of median urine protein for communities with MAR-water access vs no access were 1.11 (95% CI: 0.92, 1.35; *p*-value: 0.277) in the full multivariable-adjusted model (Table 2).

**Table 2.** Intention-to-treat effects of access to managed aquifer recharge water on blood pressure and urine total protein

Outcomes	Model 1		Model 2		Model 3	
	Regression coefficient^a^ (95% CI)	*p*-value	Regression coefficient^a^ (95% CI)	*p*-value	Regression coefficient^a^ (95% CI)	*p*-value
**All participants (*N* = 2911 person-visits for managed aquifer recharge water access and *N* = 2834 for no access)**						
Systolic BP in mmHg (mean difference)	1.96 (0.65, 3.28)	0.003	1.94 (0.63, 3.25)	0.004	1.96 (0.66, 3.26)	0.003
Diastolic BP in mmHg (mean difference)	1.46 (0.41, 2.51)	0.006	1.43 (0.38, 2.49)	0.008	1.44 (0.40, 2.48)	0.007
Mean arterial pressure in mmHg (mean difference)	1.63 (0.52, 2.74)	0.004	1.60 (0.49, 2.71)	0.005	1.62 (0.52, 2.71)	0.004
Pulse pressure in mmHg (mean difference)	0.50 (−0.07, 1.07)	0.087	0.50 (−0.04, 1.05)	0.070	0.53 (−0.02, 1.07)	0.057
Urinary total protein^b^ (ratio of medians)	1.10 (0.91, 1.33)	0.337	1.10 (0.91, 1.33)	0.326	1.11 (0.92, 1.35)	0.277
**Analyses restricted among non-hypertensive, non-diabetic, non-pregnant and no-kidney-disease participants (*N* = 2301 person-visits for managed aquifer recharge water access and *N* = 2220 for no access)**						
Systolic BP in mmHg (mean difference)	1.38 (0.01, 2.75)	0.049	1.40 (0.00, 2.80)	0.050	1.41 (0.01, 2.80)	0.048
Diastolic BP in mmHg (mean difference)	1.34 (0.22, 2.46)	0.019	1.33 (0.21, 2.45)	0.020	1.32 (0.21, 2.44)	0.020
Mean arterial pressure in mmHg (mean difference)	1.35 (0.18, 2.53)	0.024	1.35 (0.17, 2.54)	0.026	1.35 (0.17, 2.53)	0.025
Pulse pressure in mmHg (mean difference)	0.05 (−0.52, 0.61)	0.871	0.08 (−0.50, 0.65)	0.796	0.10 (−0.46, 0.67)	0.723
Urinary total protein^b^ (ratio of medians)	1.10 (0.91, 1.34)	0.337	1.11 (0.91, 1.35)	0.305	1.11 (0.92, 1.35)	0.276

a Refers to difference in mean blood pressure of participants or ratio of medians of 24-hour urinary protein of person-visits between communities with access to managed aquifer recharge water and without access.

b Analyses restricted to complete 24-hour urine samples only.

*N* = 1085 for person-visits from the access to managed aquifer recharge water group. *N* = 1060 for person-visits from the no access to managed aquifer recharge water group (reference).

Model 1: adjusted for visit only; Model 2: adjusted for visit, age, sex and body mass index; Model 3: adjusted for age, sex, body mass index, marital status, physical activity, smoking status, alcohol consumption, hours of sleep, religion, salt intake and wealth quintile.

In the restricted analyses among non-hypertensive, non-diabetic, non-pregnant participants with no history of chronic kidney disease (*N* = 4521 person-visits), we found that communities randomized to have access to MAR water had 1.41 (95% CI: 0.01, 2.80; *p*-value: 0.048) mmHg higher mean systolic BP and 1.32 (95% CI: 0.21, 2.44; *p*-value: 0.020) mmHg higher mean diastolic BP compared with communities randomized to have no access in the full multivariable-adjusted model (Table 2).

We obtained a total of 2131 complete 24-hour urine samples. Person-visits from communities with access to MAR water had 10.34 (95% CI: 1.11 19.58) mmol higher mean daily urinary Na^+^ and 2.37 (0.24, 4.49) mmol higher urinary K^+^ compared with person-visits from communities without access to MAR water in the intention-to-treat analyses (Table 3). However, there were no significant differences in the median urinary Ca^2+^ and Mg^2+^ excretion (Table 3).

**Table 3.** Urinary excretion of Na, K, Ca and Mg across different managed aquifer recharge users for intention-to-treat analyses, restricted to participants with complete 24-hour urine samples

Urinary minerals	Models	Intention-to-treat analyses		*p*-value
		No managed aquifer recharge water access	Managed aquifer recharge water access [β^a^ (95% CI)]	
		(*N* = 1048)	(*N* = 1083)	
Urinary Na^+^ (difference in mean daily excretion in mmol)	Model 1	Ref.	10.31 (0.77, 19.84)	0.034
	Model 2	Ref.	10.65 (1.44, 19.87)	0.023
	Model 3	Ref.	10.34 (1.11, 19.58)	0.028
Urinary K^+^ (difference in mean daily excretion in mmol)	Model 1	Ref.	2.38 (0.28, 4.47)	0.026
	Model 2	Ref.	2.47 (0.31, 4.63)	0.025
	Model 3	Ref.	2.37 (0.24, 4.49)	0.029
Urinary Ca^2+^ (ratio of median daily excretion)	Model 1	Ref.	1.11 (0.93, 1.34)	0.239
	Model 2	Ref.	1.12 (0.93, 1.35)	0.224
	Model 3	Ref.	1.12 (0.95, 1.33)	0.190
Urinary Mg^2+^ (ratio of median daily excretion)	Model 1	Ref.	1.05 (0.90, 1.21)	0.543
	Model 2	Ref.	1.06 (0.91, 1.23)	0.437
	Model 3	Ref.	1.06 (0.92, 1.23)	0.412

Model 1: adjusted for visit only; Model 2: adjusted for visit, age, sex and body mass index; Model 3: adjusted for age, sex, body mass index, marital status, physical activity, smoking status, alcohol consumption, hours of sleep, religion, salt intake and wealth quintile.

a Refers to differences in mean blood pressure (in mmHg) of participants or ratio of medians of 24-hour urinary protein of participants between communities with access to managed aquifer recharge water and without managed aquifer recharge water access.

Compared with groundwater-drinkers, MAR-water-drinkers had –0.24 (95% CI: –1.44, 0.95) mmHg change in mean systolic BP, –0.43 (95% CI: –1.11, 0.26) mmHg change in diastolic BP in the full multivariable-adjusted model and 0.89 (95% CI: 0.78, 1.02) ratio of medians for urine protein in the full multivariable-adjusted model (Table 4).

**Table 4.** Effect of drinking managed aquifer recharge water on blood pressure and urine protein among study participants compared with brackish groundwater-drinkers

Outcomes	Model 1		Model 2		Model 3	
	Regression coefficient^a^ (95% CI)	*p*-value	Regression coefficient^a^ (95% CI)	*p*-value	Regression coefficient^a^ (95% CI)	*p*-value
Systolic BP in mmHg (mean difference)	–0.05 (–1.24, 1.14)	0.932	–0.09 (–1.26, 1.08)	0.880	–0.24 (–1.44, 0.95)	0.690
Diastolic BP in mmHg (mean difference)	–0.24 (–0.91, 0.43)	0.487	–0.31 (–1.00, 0.37)	0.368	–0.43 (–1.11, 0.26)	0.222
Mean arterial pressure in mmHg (mean difference)	–0.19 (–0.99, 0.62)	0.652	–0.24 (–1.04, 0.56)	0.557	–0.36 (–1.17, 0.45)	0.384
Pulse pressure in mmHg (mean difference)	0.26 (–0.43, 0.96)	0.457	0.26 (–0.40, 0.93)	0.439	0.17 (–0.52, 0.86)	0.630
Urinary total protein (ratio of medians)	0.90 (0.78, 1.04)	0.154	0.89 (0.78, 1.03)	0.114	0.89 (0.78, 1.02)	0.100

a Refers to difference in mean blood pressure of participants or ratio of medians of 24-hour urinary protein of participants between communities with access to managed aquifer recharge water and without access.

*N* = 1891 person-visits for managed aquifer recharge water access group and *N* = 695 person-visits for brackish groundwater group

Model 1: adjusted for visit only; Model 2: adjusted for visit, age, sex and body mass index; Model 3: adjusted for age, sex, body mass index, marital status, physical activity, smoking status, alcohol consumption, hours of sleep, religion, salt intake and wealth quintile.

The mean systolic and diastolic BP of the three communities that did not receive MAR-water access as per randomization due to the ongoing groundwater-quality problems had somewhat higher systolic BP (113.6 mmHg in the last visit) compared with the communities that received scheduled MAR-water access (109.5 mmHg in the last visit) (Supplementar*y* Table 1, available as Supplementary data at *IJE* online). In sensitivity analyses, participants from MAR-water-access communities had 1.33 (95% CI: 0.32, 2.34; *p*-value: 0.010) mmHg higher mean systolic BP and 0.64 (95% CI: –0.20, 1.48; *p*-value: 0.136) mmHg change in mean diastolic BP compared with participants from communities without MAR-water access (Supplementar*y* Table 2, available as Supplementary data at *IJE* online). Our estimates for intention-to-treat did not change when age and BMI was used as restricted cubic splines and models were additionally adjusted for antihypertensive medications (Supplementar*y* Table 3, available as Supplementary data at *IJE* online).

## Discussion

MAR systems did not reduce BP or proteinuria in settings where alternative, lower-salinity water was available. Moreover, few households consumed the MAR water. Contrary to our hypothesis, the intention-to-treat analyses suggested that access to MAR water increased the mean study-population BP. Pond water was the most common counterfactual water source and had consistently lower salinity than the MAR water across all visits. Access to MAR water, therefore, exposed communities to higher-salinity drinking and cooking water and to higher sodium intake (evident from the estimated sodium intake of different water sources) compared with drinking counterfactual pond water. Urine mineral analyses suggest that communities with MAR-water access had a significantly higher mean daily urinary sodium demonstrating that the intervention failed to reduce the ceiling exposure in the population and so explains the higher BP among participants from MAR communities in intention-to-treat analyses.

Despite the promotion of MAR water by CHPs, we found poor adherence to water consumption from MAR systems, particularly for cooking purposes. Water, sanitation, and hygiene (WASH) interventions have demonstrated low uptake in effectiveness trials and routine programmes implemented at scale.^33–35^ In terms of adopting a new behaviour, when options are available, people usually choose convenience over effectiveness.^36^ For instance, several household-based filters to provide low-arsenic drinking water in Bangladesh had lower uptake. Studies found affordability, convenience of use and maintenance, and availability of alternate water sources are important determinants for the acceptability of water arsenic-removal interventions.^37–39^ Household-based arsenic-removal filters had a lower acceptability by the users due to recurrent cost and complicated maintenance procedures.^39^^,^^40^ In contrast, low-arsenic-containing deep tube wells were well accepted by Bangladeshi communities due to easy operation, year-round availability of water and negligible maintenance procedures.^37^^,^^38^

Women are mostly responsible for household water collection and cooking in Bangladesh,^41^ and they may find it difficult to collect water from a distant MAR system that is accessible for limited hours of the day compared with a nearby pond-water source. A qualitative study conducted in south-west coastal Bangladesh found that some consumers expressed dissatisfaction with poor management and maintenance of MAR systems, financial cost, poor MAR-water quality and the time required to collect MAR water.^42^ Low adherence can also be explained by the preference for lower-salinity pond water over MAR water. Unpalatable taste in other contexts in Bangladesh have affected uptake.^43^

MAR systems are successful at reducing aquifer salinity.^14^ Our data also suggest that the electrical conductivity of MAR water was consistently lower than that of the brackish groundwater. South-west coastal Bangladesh often faces inundation by seawater due to cyclone-associated tidal surges. Two recent cyclones that rampaged the ‘Sidr’ region in 2007 caused a 5- to 6-metre tidal surge and cyclone ‘Aila’ in 2009 caused a 3-metre surge, both affecting the study communities.^44^ Ponds were inundated by seawater during these cyclones and were not suitable for consumption for multiple years.^45^ These cyclones highlighted the need for more reliable and disaster-resilient drinking-water sources—a need that motivated the construction of the 20 MAR systems that were first piloted in 2009.^46^ The additional 80 MAR sites were constructed during 2012–2014 and represented a strategy for increasing low-salinity-water availability and sustaining a year-round drinking-water supply, since freshwater infiltration and storage are done at the groundwater level. Whereas many of these MAR systems have achieved their technical objectives, several years of rainfall after 2009 reduced salinity in the ponds that were inundated by cyclones and communities have resumed the consumption of pond water.^47^ Future cyclones may again inundate ponds and communities may then find MAR water a preferable water source. Another possible benefit of the MAR system is the improved microbiological quality of MAR water over the pond water.^48^ MAR systems filter the pond water when it passes through the sand and gravel layers of the MAR infiltration wells.^14^

There are limitations to our study. First, we did not recognize that communities were consuming pond water instead of brackish groundwater during the dry season before the commencement of our study. Prior studies from south-west coastal Bangladesh reported that communities drinking groundwater had high BP and pregnant women drinking groundwater had a higher prevalence of pre-eclampsia.^17^^,^^18^ These studies also reported that study participants consumed groundwater (tube well) as the predominant drinking-water source in the region. There may be concerns about whether conducting a MAR health evaluation was appropriate given that the communities were not using brackish groundwater. Nevertheless, this was an evaluation of an otherwise-advocated intervention. Without this evaluation, we would not have identified that communities were exposed to high-sodium-containing water by providing access to MAR water.

We may have found that access to MAR water lowered BP if the non-intervention counterfactual households consumed brackish groundwater. *Post hoc* restricted analyses comparing MAR-water-drinkers and brackish groundwater-drinkers suggested that MAR-water-drinkers did not have increased BP and proteinuria compared with groundwater-drinkers but, with the limited sample size, these differences may also have been due to chance. Three communities did not receive MAR access per the randomization schedule due to technical issues, including the presence of sand in the water. These three were included as intervention communities in the intention-to-treat analyses and may have introduced differential misclassification bias. We think this would induce a positive bias away from the null, since these three communities had somewhat higher mean BP compared with the other intervention communities. We observed heterogeneity in the mean BP across communities during baseline and considering these three sites as non-MAR communities in sensitivity analyses attenuated the intention-to-treat (ITT) findings. Nevertheless, this did not change our inference, as systolic BP remained significantly higher in the communities with MAR-water access compared with communities without MAR-water access. We did not collect information about whether participants had consumed water from other sources, particularly when they were outside the home. We also did not collect data on dietary mineral intake through food that is likely to be different across participants. Nevertheless, we think the lack of this unmeasured information is unlikely to have affected our estimates, for two reasons: (i) participant-level random effects may have largely captured these habitual unmeasured characteristics and (ii) each participant contributed data to both the intervention and control times in the stepped-wedge design. The estimates of intention-to-treat analyses were not affected by different levels of confounder adjustments (Table 2). There was potential for urine-biomarker measurement error: 24-hour urine biomarkers can be misleading if the urine samples were truly collected over periods differing from 24 hours (“over-collection” or “under-collection”).^28^ We therefore restricted the analyses to participants who had complete 24 hour samples, in models where outcomes were urine biomarkers.

Although MAR systems reduced the salinity of the brackish aquifer, consumption of MAR water neither reduce BP nor proteinuria during the study period when there was no cyclone and storm surge. MAR systems may have limited acceptability in communities where alternative low-salinity water sources such as rainwater and pond water are available. Considering the high risk of sodium intake and high BP, we do not support the scale-up of the MAR systems as a routine drinking-water source in south-west coastal Bangladesh if communities have available low-salinity pond water. Nevertheless, this paper does not report the effectiveness of the MAR system to improve the microbiological water quality or evaluation of MAR systems as a climate-resilient drinking water source. Our findings highlight that community reliance on a specific water source may change over time and thus the future evaluation of drinking water salinity-lowering interventions should carefully assess the community water-consumption practices.

## Supplementary data

Supplementary data are available at *IJE* online.

## Author contributions

A.M.N., L.U., M.R., K.M.A., T.F.C., and S.P.L. designed the study. A.M.N. drafted the research protocol and manuscript with the feedback from all listed co-authors. A.M.N., S.D., M.R., and L.U. oversaw the study implementation and coordinated the data collection and laboratory analyses of the environmental and human samples. S.P.L., T.F.C., and M.O.G. provided scientific and epidemiological input during implementation of the study. K.M.A., and M.S. provided hydro-geological input during protocol development and study implementation. M.O.G., A.M.N., and H.C. developed the analytical approach, A.M.N. conducted the statistical analysis and constructed the tables and figures. All authors reviewed the manuscript.

## Supplementary Material

dyaa098_Supplementary_Data
