# Supplementary material for: Consequences of access to water from managed aquifer recharge systems for blood pressure and proteinuria in south-west coastal Bangladesh: a stepped-wedge cluster-randomized trial
Source: Int J Epidemiol. 2020 Jul 12;50(3):916–28. doi: 10.1093/ije/dyaa098 (PMC8271187; doi:10.1093/ije/dyaa098)
Supplement: dyaa098_Supplementary_Data [file dyaa098_supplementary_data.doc]

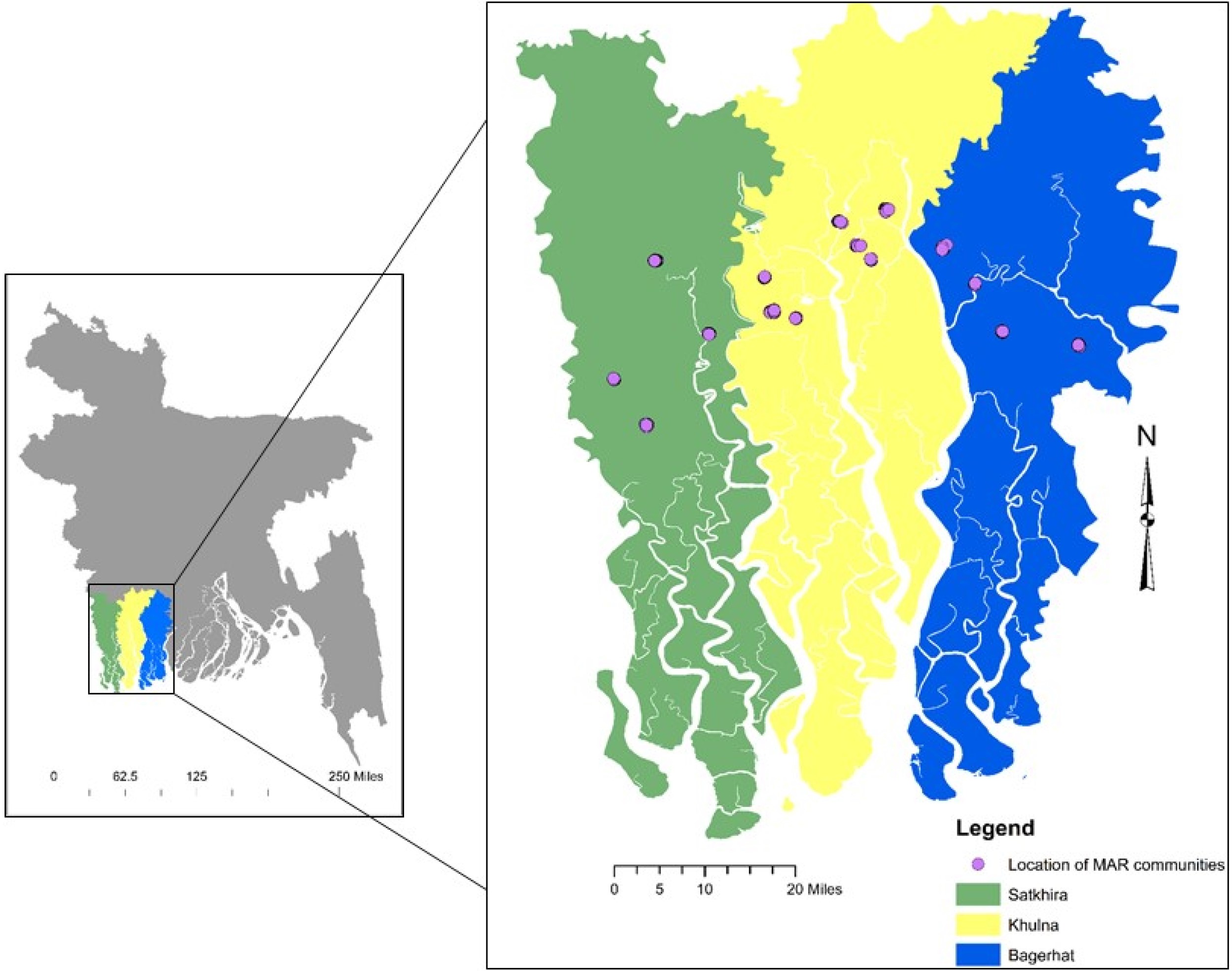


**Supplementary Figure S1: Managed aquifer recharge study sites in southwest coastal Bangladesh**


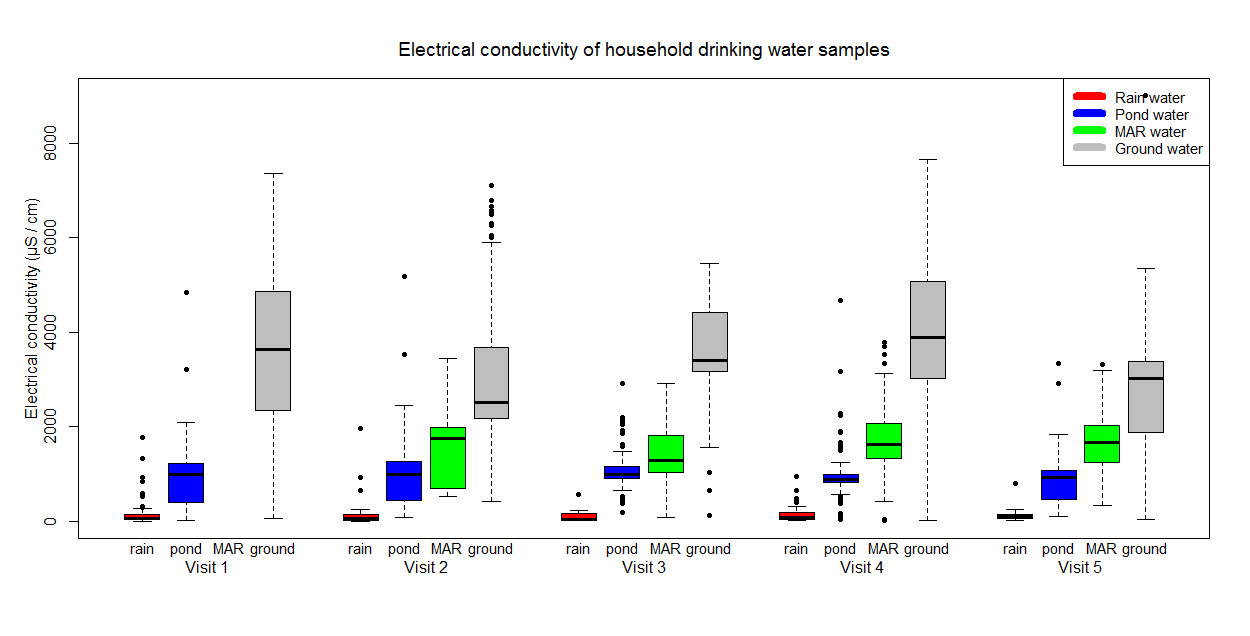


**Supplementar*y* Figure S2: Electrical conductivity of the households' stored drinking water samples across visits. Electrical conductivity is the unity of salinity that represents all dissolved ions in water. Electrical conductivity was measured at field level using Hanna Salinity™ meter.**

| **Supplementar*y* Table S1: Mean blood pressure in communities included in intention-to-treat-analyses from the managed aquifer recharge (MAR) trial.** | | | | |  |  |
| --- | --- | --- | --- | --- | --- | --- |
| Blood pressure | Visit 3 | | Visit 4 | | Visit 5 | |
|  | Received MAR access as scheduled (n=7) | Did not received MAR access as scheduled^✝^ (n=1) | Received MAR access as scheduled (n=10) | Did not received MAR access as scheduled^✝^ (n=2) | Received MAR access as scheduled (n=13) | Did not received MAR access as scheduled^✝^ (n=3) |
| Systolic BP (mmHg) | 112.7 | 115.2 | 110.1 | 112.4 | 109.5 | 113.6 |
| Diastolic BP (mmHg) | 66.4 | 68.2 | 65.2 | 66.9 | 65.3 | 68.7 |
| ^✝^Access was not provided due to technical issues (e.g. presence of sand in MAR water) | | | | | | |

**Supplementary Table S2: Sensitivity analyses for the effects of access to managed aquifer recharge (MAR) water on blood pressure and urine protein. N=2495 person-visits for access to MAR water group and N=3259 person-visits no MAR water access group (reference). The three communities without MAR water access as per randomization schedule were considered in no MAR water access group.**

| **Outcomes** | **Model 1** | | | **Model 2** | | | **Model 3** | |
| --- | --- | --- | --- | --- | --- | --- | --- | --- |
|  | Regression coefficient ^*^  (95% CI) | p-value | Regression coefficient ^*^  (95% CI) | | p-value | Regression coefficient ^*^  (95% CI) | | p-value |
| Systolic BP in mmHg (mean difference) | 1.28 (0.26, 2.29) | 0.014 | 1.31 (0.29, 2.33) | | 0.011 | 1.33 (0.32, 2.34) | | 0.010 |
| Diastolic BP in mmHg (mean difference) | 0.64 (-0.20, 1.49) | 0.137 | 0.64 (-0.22, 1.50) | | 0.149 | 0.64 (-0.20, 1.48) | | 0.136 |
| Mean arterial pressure in mmHg (mean difference) | 0.86 (0.00, 1.72) | 0.051 | 0.87 (-0.01, 1.74) | | 0.053 | 0.88 (0.02, 1.73) | | 0.046 |
| Pulse pressure in mmHg (mean difference) | 0.60 (0.03, 1.16) | 0.038 | 0.65 (0.10, 1.19) | | 0.021 | 0.68 (0.14, 1.23) | | 0.014 |
| Urinary total protein (ratio of medians) | 1.10 (0.91, 1.34) | 0.321 | 1.11 (0.91, 1.34) | | 0.309 | 1.12 (0.92, 1.35) | | 0.266 |

^*^ Refers to difference in mean blood pressure of participants or ratio of medians of 24-hour urinary protein of participants between communities with access to MAR water and without access.

Model 1: Adjusted for visit only; Model 2: Adjusted for visit, age, sex and body mass index

Model 3: Adjusted for age, sex, body mass index, marital status, physical activity, smoking status, alcohol consumption, hours of sleep, religion, salt intake and wealth quintile

**Supplementary Table S3: Intention-to-treat effects of access to managed aquifer recharge (MAR) water on systolic and diastolic blood pressure, when restricted cubic splines of age and BMI were used in the model and models were additionally adjusted for the use of anti-hypertensive medications.**

| **Outcomes** | **Model 2** | | | **Model 3** | | | **Model 4** | |
| --- | --- | --- | --- | --- | --- | --- | --- | --- |
|  | Regression coefficient ^*^  (95% CI) | p-value | Regression coefficient ^*^  (95% CI) | | p-value | Regression coefficient ^*^  (95% CI) | | p-value |
| Systolic BP in mmHg (mean difference) | 1.94 (0.64, 3.24) | 0.003 | 1.98 (0.68, 3.28) | | 0.003 | 1.99 (0.69, 3.30) | | 0.003 |
| Diastolic BP in mmHg (mean difference) | 1.43 (0.38, 2.48) | 0.008 | 1.45 (0.42, 2.48) | | 0.006 | 1.46 (0.43, 2.49) | | 0.006 |

^*^ Refers to difference in mean blood pressure of participants between communities with access to MAR water and without access.

Model 2: Adjusted for visit, age, sex and body mass index

Model 3: Adjusted for age, sex, body mass index, marital status, physical activity, smoking status, alcohol consumption, hours of sleep, religion, salt intake and wealth quintile

Model 4: Adjusted for age, sex, body mass index, marital status, physical activity, smoking status, alcohol consumption, hours of sleep, religion, salt intake, wealth quintile, and use of anti-hypertensive medication
